# A Pilot Study of Anti-CTLA4 Antibody Ipilimumab in Patients with Synovial Sarcoma

**DOI:** 10.1155/2013/168145

**Published:** 2013-02-27

**Authors:** Robert G. Maki, Achim A. Jungbluth, Sacha Gnjatic, Gary K. Schwartz, David R. D'Adamo, Mary Louise Keohan, Michael J. Wagner, Kelly Scheu, Rita Chiu, Erika Ritter, Jennifer Kachel, Israel Lowy, Lloyd J. Old, Gerd Ritter

**Affiliations:** ^1^Department of Medicine, Memorial Sloan-Kettering Cancer Center, New York, NY 10065, USA; ^2^Departments of Medicine, Pediatrics, and Orthopaedics, Mount Sinai School of Medicine, 1 Gustave L. Levy Place, P.O. Box 1208, New York, NY 10029-6574, USA; ^3^Ludwig Institute of Cancer Research, New York, NY 10065, USA; ^4^Sarcoma and Bone Cancer Treatment Center, Dana-Farber Cancer Institute, Boston, MA 02215, USA; ^5^University of Michigan Cancer Center, Ann Arbor, MI 48109, USA; ^6^Medarex, Inc., Bloomsbury, NJ 08804, USA; ^7^Bristol Myers Squibb Company, Wallingford, CT 06492, USA; ^8^Regeneron, Inc., Tarrytown, NY 10591, USA

## Abstract

*Background*. Patients with recurrent synovial sarcomas have few options for systemic therapy. Since they express large amounts of endogenous CT (cancer testis) antigens such as NY-ESO-1, we investigated the clinical activity of single agent anti-CTLA4 antibody ipilimumab in patients with advanced or metastatic synovial sarcoma. *Methods*. A Simon two-stage phase II design was used to determine if there was sufficient activity to pursue further. The primary endpoint was tumor response rate by RECIST 1.0. Patients were treated with ipilimumab 3 mg/kg intravenously every 3 weeks for three cycles and then restaged. Retreatment was possible for patients receiving an extra three-week break from therapy. Sera and peripheral blood mononuclear cells were collected before and during therapy to assess NY-ESO-1-specific immunity. *Results*. Six patients were enrolled and received 1–3 cycles of ipilimumab. All patients showed clinical or radiological evidence of disease progression after no more than three cycles of therapy, for a RECIST response rate of 0%. The study was stopped for slow accrual, lack of activity, and lack of immune response. There was no evidence of clinically significant either serologic or delayed type hypersensitivity responses to NY-ESO-1 before or after therapy. *Conclusion*. Despite high expression of CT antigens by synovial sarcomas of patients treated in this study, there was neither clinical benefit nor evidence of anti-CT antigen serological responses. Assessment of the ability of synovial sarcoma cell lines to present cancer-germ cell antigens may be useful in determining the reason for the observed lack of immunological or clinical activity.

## 1. Introduction

Synovial sarcoma (SS) is one of the more common forms of soft tissue sarcoma, with an incidence of approximately 3 per million [1]. It presents as a painless or painful mass, typically in an extremity, with a peak incidence around adolescence and young adulthood [2]. SS is characterized genetically by a t(X;18) translocation involving *SS18* and either *SSX1*,* SSX2*,or* SSX4 *[3–8]. While surgery and radiation therapy are usually effective in controlling local disease [9], approximately 40% of patients with synovial sarcoma will die of metastatic disease [10]. Chemotherapies including ifosfamide, doxorubicin, and possibly trabectedin have demonstrated activity against SS; however, responses are not durable [11–14]. As a result, the development of novel therapies remains a focus of research for this diagnosis.

One area of focus has centered on the finding that the *SS18-SSX* translocation product is associated with the upregulation of a variety of cancer-testis antigens (CTAs), such as some antigens of the *MAGE-A *family and *NY-ESO-1*, which are generally found in normal adult tissues solely in testicular germ cells and occasionally in placenta. However, CTAs are upregulated in a variety of malignant tumors [15]. Interestingly, the *SSX* genes represent CTAs as well [16]. In comparison to other malignant tumors, there is a high incidence of CTA expression in synovial sarcoma; moreover, the expression pattern is highly homogeneous as opposed to a heterogeneous pattern in most other tumors [17–19]. Furthermore, immune responses, in particular antibody responses and T-cell responses to certain CTAs, can be detected in patients with a variety of CTA-expressing tumors [20–22]. 

These data were the basis of clinical trials that demonstrated impressive responses of tumors to various immune mediated therapies. Notably, Hodi et al. showed that ipilimumab improved survival in patients with metastatic melanoma [23]. This response is perhaps seen because ipilimumab enhances immunity against CTAs [24, 25]. Similar responses were seen with infusion of genetically engineered lymphocytes reactive with NY-ESO-1 in melanoma patients [26]. This latter study also demonstrated objective clinical responses in 4 of 6 patients with SS. Recent data indicate the activity of immune costimulator anti-PD1 in several solid tumor subtypes [27]. 

Given the high incidence and homogeneous expression of CTAs in SS, we conducted a phase II study of anti-CTLA4 antibody ipilimumab as a means to increase endogenous T-cell responses against CTAs, with the hope of engendering a radiological and/or clinical response. We describe herein the results of the study, which was terminated early due to slow accrual, lack of clinical efficacy, and lack of immune response in the first six patients treated on study. 

## 2. Methods

This was a single cohort, single center, and open label phase II study of ipilimumab in patients with advanced synovial sarcomas. Institutional review board approval of the protocol had been granted to perform the study. Each participant provided written informed consent. RECIST response determinations were made by study radiologists; images were not reviewed centrally. Radiology results were subject to confirmatory review by an independent committee at Memorial Hospital. Death data were obtained using the Social Security Death Index.

### 2.1. Study Design

The primary objective of the study was to determine the radiological response rate of patients with advanced synovial sarcoma following treatment with ipilimumab, as per Response Evaluation Criteria in Solid Tumors (RECIST) 1.0 definitions. The secondary objectives of the study were to (1) determine the clinical benefit rate (CR + PR + stable disease (SD)) of patients with advanced synovial sarcoma following treatment with ipilimumab, (2) evaluate NY-ESO-1 specific immunity (NY-ESO-1 and LAGE-1 antibody, CD8+ and CD4+ T cells, and delayed-type hypersensitivity (DTH)) induced by three doses of ipilimumab in this patient population, (3) and determine the safety of ipilimumab in this group of patients.

The study was designed as a Simon two-stage phase II study [28]. The outcome representing futility was a 5% RECIST response rate, and indicator of activity was a 25% RECIST response rate. For an alpha of 0.05 and power (1-beta) of 80%, the study was to be stopped if there were no responses after 9 patients were accrued. If there was at least one RECIST partial response (PR), another 8 patients were to be accrued for a total of 17 patients. The drug would be declared inactive if there were 2/17 or fewer responses and declared worthy of further investigation if there were at least 3/17 RECIST PR. With this design, there was a 63% probability of study termination after 9 patients if the true response rate had been 5%.

### 2.2. Ipilimumab Administration

Three doses of ipilimumab, 3 mg/kg over 90 minutes each at 1 mL/min, were administered by intravenous infusion at 3-week intervals. Premedication was not given with the first dose of therapy. The agent was supplied by Medarex, Inc. A 6-week observation period followed the final dose. Toxicity and immunological assessments were made throughout the 12 week study period. In the absence of disease progression (requiring other treatment) or grade 3 or greater toxicity, patients were eligible to receive an additional three doses of anti-CTLA4 following the same schedule as the first 12 weeks of treatment. 

### 2.3. Eligibility

Entry criteria included the following.

*Inclusion Criteria*. Locally recurrent or metastatic synovial sarcoma with RECIST defined measurable disease, who failed or refused standard treatment; Eastern Cooperative Oncology Group (ECOG) performance status 0–2; laboratory constraints: absolute neutrophil count ≥1.0 × 10^9^/L; hemoglobin ≥8 g/dL; platelet count ≥75 × 10^9^/L; serum creatinine ≤2 mg/dL; ALT, AST ≤ 5 × institutional upper limit of normal (ULN); alkaline phosphatase, total bilirubin ≤ 2.5 × ULN.

*Exclusion Criteria*. Clinically significant heart disease (NYHA Class III or IV), other serious illnesses, or intercurrent illness, requiring hospitalization; patients with a second cancer diagnosis in the last 5 years, except for basal cell carcinoma, completely resected, or cervical carcinoma in situ, completely resected; known HIV positivity or history of autoimmune disease; chronic use of immunosuppressive drugs such as systemic corticosteroids; women who were pregnant or breast feeding; refusal or inability to use effective means of contraception (all men and women with childbearing potential).

### 2.4. NY-ESO-1 Protein Expression

In-situ presence of NY-ESO-1 was analyzed by immunohistochemistry as previously described [17]. Briefly, analysis was done on representative tumor sections of archival formalin-fixed paraffin-embedded tissue blocks. For the detection of NY-ESO-1, monoclonal antibody E978 was used in combination with a heat-based antigen retrieval method and a Powervision secondary kit (Leica Biosystems, Wetzlaer, Germany). Diaminobenzidine (Liquid DAB, Bioegenx, San Ramon) was used as a chromogen, and testicular tissue with preserved spermiogenesis was used as a positive control.

### 2.5. Correlative Studies

HLA typing was performed in all patients. Tumor typing for NY-ESO-1 was performed by RT-PCR and/or immunohistochemistry. Humoral and cellular immunity were assayed at different times before and after treatment as described previously and included (1) enzyme-linked immunosorbent assay (ELISA) tests for anti-NY-ESO-1 and anti-LAGE-1 from serum (see the following); (2) total T-cell number (CD3+ T-cell count); (3) ELISPOT assays for NY-ESO-1-specific T cells: Ad2/NY-ESO-1; HLA-A2/NY-ESO-1b; HLA-DP4/NY-ESO-1-DP4; and (4) tetramer staining of T cells (peripheral blood NY-ESO-1b specific CD8+ T cells in HLA-A∗0201(+) patients). All patients underwent delayed type hypersensitivity (DTH) skin testing with NY-ESO-1 protein, NY-ESO-1b, and DP4 peptides and had analysis for ANA and human anti-human antibody production (HAHA). Indirect ophthalmoscopy and slit lamp examination were performed before and after 3 cycles of therapy to rule out the possibility of uveitis on ipilimumab. 

### 2.6. Enzyme-Linked Immunosorbent Assay (ELISA)

Patient serum samples were analyzed by ELISA for seroreactivity to various recombinant proteins, including NY-ESO-1. Sera were serially diluted starting from 1 : 100 and added to low-volume 96-well plates (Corning) coated with 1 *μ*g/mL antigen and blocked with PBS containing 5% nonfat milk. After incubation, plates were washed with PBS containing 0.2% Tween and rinsed with PBS. Sera IgG bound to antigens was detected with an alkaline phosphatase conjugated specific mAb (Southern Biotech). After addition of ATTOPHOS substrate (Fisher Scientific), absorbance was measured using a fluorescence reader Cytofluor Series 4000 (PerSeptive Biosystems). A reciprocal titer was calculated for each serum sample as the maximal dilution still significantly reacting to a specific antigen. Specificity was determined by comparing seroreactivity among various antigens tested. In each assay, sera of patients with known presence or absence of specific reactivity were used as controls. A positive result was defined as extrapolated reciprocal titers of >100.

### 2.7. Statistical Methods

Given the small number of correlative study samples, statistics were descriptive only.

## 3. Results

### 3.1. Demographics

Six patients (4 women, 2 men) were enrolled between June 2005 and April 2007. All patients were Caucasian. All patients had failed doxorubicin and ifosfamide-based therapy before enrolling on study. After failing treatment on this study, three patients received no further chemotherapy, while for the other three, treatment of an unknown type was delivered through local facilities. No data are available on the agents employed. Their HLA status regarding HLA-A2 and HLA-DP4 is indicated in Table 1. All tumors expressed NY-ESO-1 protein (from 2+ to 4+) by immunohistochemistry [17]. 

### 3.2. Toxicity, Dose Reductions

A total of 51 adverse events were reported for the six patients on study. The adverse events and their relationship to study medication are indicated in Table 2. There were 2 reported serious adverse events (SAE). One patient had severe vomiting and diarrhea, considered possibly related to ipilimumab. A report of dyspnea was considered unrelated to study drug. No dose reductions were employed in the study.

### 3.3. Clinical Outcomes

All were taken off study due to progressive disease after the first cycle of therapy. Four completed all three of the planned doses of ipilimumab: one patient received two, and one patient received one dose of therapy before developing radiological and/or clinical progression. Time to progression ranged from 0.47 to 2.1 months (median 1.85), and overall survival times ranged from 0.77 to 19.7 months (median 8.75 months).

### 3.4. Translational Studies

Patient sera showed no clear evidence of anti-NY-ESO-1 or anti-LAGE antibodies before or after initiation of therapy with ipilimumab. As a result, ELISPOT assays against NY-ESO-1 HLA class I and class II peptides were not pursued.

No patient had a DTH reaction to intact NY-ESO-1 protein, NY-ESO-1b HLA class I, or NY-ESO-1-DP4 HLA class II peptides. There was no evidence in patients of either ANA antibodies or HAHA before or after the start of treatment with ipilimumab, and no patient had any evidence of uveitis on ophthalmologic examination.

**Figure 1:**
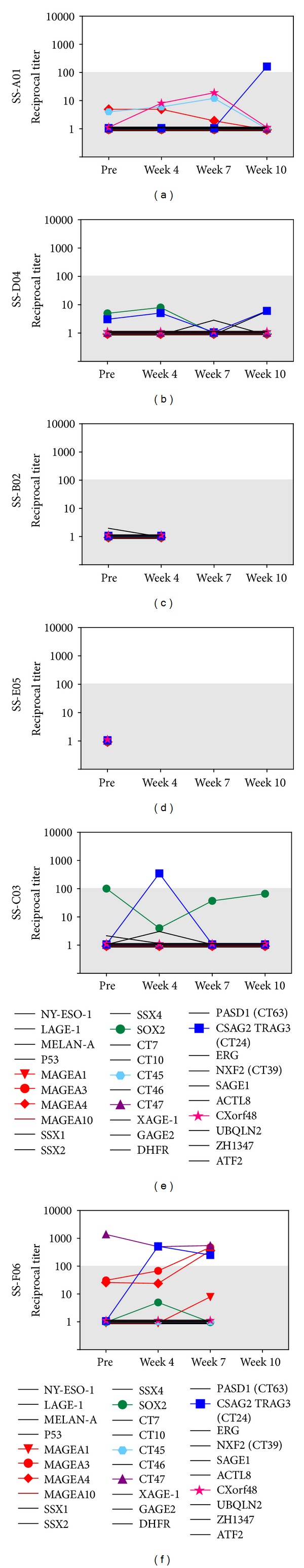
One study patient demonstrated an immune response to a CTA after ipilimumab administration. Plots demonstrating immune titers in each patient to the specified CTA before ipilimumab and at weeks 4, 7, and 10 are presented. Only patient SS-F06 reproducibly showed a weak preexisting serum antibody response to MAGEA3 and MAGEA4, that increased after treatment. Patient SS-F06 also had serum antibodies to CT47 throughout the study that did not change in titers and seroconversion against CSAG2 after the first ipilimumab injection. Patient SS-C03 had some reactivity to SOX2 and had a transient response to CSAG2.

One patient reproducibly showed a weak preexisting serum antibody response to MAGEA3 and MAGEA4 that increased after treatment (Figure 1). The same patient also had serum antibodies to CT47 throughout the study that did not change in titers, and a seroconversion against CSAG2, a new CT antigen not well characterized, observed after the first ipilimumab injection. A second patient had some reactivity to SOX2 that did not change with treatment. This patient also demonstrated a transient response to CSAG2 only seen at week 4. 

## 4. Discussion

Clinical trials have demonstrated the efficacy of immunomodulating treatments in various cancer types, including SS. Two randomized phase III trials showed a modest, but significant, response of melanoma to CTLA-4 antagonist ipilimumab [23, 29]. Ipilimumab appears to act at least in part by enhancing cellular immunity to NY-ESO-1. This is further supported by the demonstration that treatment with autologous T cells engineered to recognize NY-ESO-1 antigen in treatment of both melanoma and SS produced striking results in some patients [26, 30]. 

We had similarly hypothesized that SS patients would benefit from immunomodulation with ipilimumab, hoping to observe radiological or immunological activity. However, due to slow accrual after rapid progression of several patients in the initial cohort of the study and lack of immune response to NY-ESO-1, this study was terminated. The positive immune responses to other CTAs that are presented here were found using reagents that were not available at the time the study was conducted. The clinical significance of these responses is currently unclear. In light of the promising data by Rosenberg and colleagues [26] and hints of activity of vaccines directed against other antigens in a series of studies in Japan [31–36], the data presented here suggest that triggering an effective anti-SS response is not accomplished by CTLA-4 inhibition alone. 

We were somewhat surprised by the lack of an immune response against NY-ESO-1 in these patients, given the high rates of CTA expression in tumors of this type [17, 19]. Interestingly, at least one previous study showed that sarcoma patients, in particular those with SS, were less likely to generate humoral antibodies to CTAs, with only 2 of 54 sarcoma patients and 0 of 5 synovial sarcoma patients demonstrating spontaneous humoral immunity to NY-ESO-1 [37]. A later study in 2004 described one SS patient with a spontaneous immune response to NY-ESO-1 [38], suggesting that the immunogenicity of sarcomas is variable. It was further suggested that immunogenicity of other cancers like melanoma and ovarian cancer is somewhat higher than what has been reported in sarcoma [20], especially considering how likely it is that sarcomas will express CTAs. 

There is a variety of reasons for the observed immune disparity between this and other clinical trials. First, the results here suggest that the transfer of autologous T-cells and immune stimulation with CTLA-4 inhibition are not equivalent strategies to trigger an anti-tumor response. As there was never any evidence of NY-ESO-1 immunologic sensitivity in any of the 6 patients in this study, factors other than CTLA-4 inhibition may be required to create an antitumor response to CTAs. 

These findings may also be explained by the insight, published after this study was completed, that RECIST is not the ideal criteria to use for assessment of tumor response with immunological agents. Responses to ipilimumab can be delayed, requiring additional assessments over a longer time period [39]. Unfortunately this assessment was not possible in this study population, as it was not possible to apply different study criteria when the patients were already off study. The short time from study discontinuation to death indicates that a late immune response would likely not have been detected. It is also important to note that the six patients in this study perhaps fared worse than the average person with this diagnosis just diagnosed with metastatic disease. For comparison, the median survival in the PALETTE phase III study was 10.7 months, compared to 8.75 months in this study [40]. Additionally, only one dose, 3 mg/kg, was used in this study. It is possible that a higher dose would elicit a NY-ESO-1 response in the study population. 

Additional studies demonstrating whether or not SS present CTAs to the immune system, in addition to merely expressing them, are one necessary component to connect this chain of data. The role of tumor infiltrating lymphocytes, clear in melanoma, is also not well examined in SS. Finally, due to the very small sample size of this trial, we may not have had sufficient power to identify what may be a small subset of patients who may benefit from ipilimumab alone. Thus, despite the overt inactivity of ipilimumab in the setting of metastatic SS, one could envision use of ipilimumab or other costimulatory antibodies such as anti-PD1 in patients receiving either vaccines or T cells directed against NY-ESO-1 or other CTAs. By publishing this negative trial we hope that similar trials in the future will not repeat our mistakes. We remain hopeful that future studies will point the way toward the appropriate use of immunotherapy for this difficult clinical situation.

## Figures and Tables

**Table 1:** Patient characteristics.

Age	Gender	NY-ESO-1 immuno-histochemistry	HLA-A2	HLA-DP4	ECOG performance status	Time to progression (months)	Overall survival (months)
43	F	3+	+	+	1	1.9	3.8
26	F	4+	+	+	1	0.9	19.7
56	M	3+	+	+	0	1.9	13.7
32	F	3+	−	+	1	2.1	3.2
57	F	3+	−	−	2	0.5	0.8
23	M	2+	+	+	1	1.8	13.7

**Table 2:** Adverse events on study and reported relationship to study drug.

System organ class	Adverse event (preferred term)	Number of events	CTC severity scale					Relationship to study drug*				
			**1**	**2**	**3**	**4**	**5**	**1**	**2**	**3**	**4**	**5**
	Total numbers	**51**	**25**	**17**	**9**			**18**	**14**	**11**	**8**	
Blood and lymphatic disorders		**2**			**2**				**2**			
	Lymphopenia	2			2				2			
Endocrine disorders		**1**		**1**					**1**			
	Hyperglycemia	1		1					1			
Gastrointestinal disorders		**12**	**5**	**2**	**5**				**1**	**3**	**8**	
	Abdominal pain	1		1					1			
	Diarrhea	4	2		2					1	3	
	Gastritis	2	1	1							2	
	Nausea	2	1		1						2	
	Proctalgia	1	1							1		
	Vomiting	2			2					1	1	
General disorders and administration site reactions		**1**	**1**							**1**		
	Fatigue	1	1							1		
Hepatobiliary disorder		**10**	**5**	**4**	**1**				**7**	**3**		
	Hyperbilirubinemia	10	5	4	1				7	3		
Infections and infestations		**1**		**1**				**1**				
	Bronchitis	1		1				1				
Investigations		**14**	**8**	**5**	**1**			**13**		**1**		
	Alanine aminotransferase	1	1					1				
	Aspartate aminotransferase	2	2					2				
	Blood creatinine	1	1					1				
	Blood alkaline phosphatase	6	4	2				6				
	Hemoglobin	1		1				1				
	Platelet count	2		1	1			2				
	White blood count	1		1						1		
Metabolism and nutrition disorders		**3**	**1**	**2**					**3**			
	Hypoalbuminemia	1		1					1			
	Hypocalcemia	1	1						1			
	Hypophosphatemia	1		1					1			
Musculoskeletal and connective tissue disorders		**2**	**2**							**2**		
	Muscular weakness	1	1							1		
	Musculoskeletal chest pain	1	1							1		
Respiratory, thoracic, and mediastinal disorders		**3**	**1**	**2**				**3**				
	Chest pain	2	1	1				2				
	Pleural effusion	1		1				1				
Skin and subcutaneous disorders		**2**	**2**					**1**		**1**		
	Dermatitis acneiform	1	1					1				
	Exfoliative rash	1	1							1		

*Relationship to study drug: 1: not related, 2: unlikely related, 3: possibly related, 4: probably related, and 5: definitely related.

## Abbreviations

SS: Synovial sarcoma
CTLA-4: Cytotoxic T-lymphocyte antigen 4
CTA: Cancer testis antigen.
